# The curative effect analysis of peripherally inserted central venous catheter catheterization for tumor patients under the guidance of new medical guide wire

**DOI:** 10.1186/s40001-021-00571-1

**Published:** 2021-08-28

**Authors:** Weizhu Xiao

**Affiliations:** Department of Blood Specialty, The Second Affiliated Hospital of Fujian Medical University, No. 34, Zhongshanbeilu, Quanzhou City, 36200 Fujian Province China

**Keywords:** Guidewire, PICC, Positioning

## Abstract

**Objective:**

To report a method of PICC catheterization with the aid of a new medical guide wire, and to discuss the curative effect.

**Methods:**

Five hundred and thirteen patients who visited our hospital from January 2013 to December 2019 for PICC catheterization were selected as study subjects. Random number method was used to group patients. General information was recorded for both groups. Patients in the observation group received catheterization with the aid of a new medical guide wire. The control group received catheterization via conventional guide wire. The success rate of the first catheterization, the success rate of the catheterization, the timing of the catheterization, the complications and the position of the catheter end were compared between the two groups.

**Results:**

There was no significant difference in general information between the two groups. After analyzing the puncture situation of the two groups, it was found that the average catheterization time of the observation group was shorter than that of the control group, and the difference was statistically significant. Patients in the observation group had higher success rate of one-time catheterization and catheterization success rate, and the difference was statistically significant. The incidences of occult thrombosis, phlebitis and catheter blockage in the observation group were lower than those in the control group, and the difference was statistically significant. The incidence of dominant thrombosis and bleeding at puncture point in the observation group was also lower than that in the control group, but the difference was not statistically significant.

**Conclusion:**

The new type of medical guide wire component is effective for PICC catheterization and worthy of further promotion.

## Introduction

Peripherally inserted central venous catheter (PICC) is one of the most common venous pathways. Many cancer patients require PICC catheterization. This approach first avoids direct contact between chemotherapeutic drugs and the upper limb veins. However, the rich blood flow of large veins can rapidly dilute drugs, thus reducing the incidence of phlebitis and improving the quality of life of patients [1]. In the past, the modified Seldinger technique (MST) with guide wire guided electrocardiography was mostly used for puncture and localization [2]. However, some patients have poor vascular conditions, so it is difficult to achieve one-off success. Some scholars have adopted ultrasound-guided localization for puncture [3], but not all hospitals have professional intravenous therapy nurses who can master ultrasonic technology, and bedside portable ultrasound machine has not been widely used in the grassroots level. In addition, to realize ECG positioning technology, professional instruments or media products such as guidewire, ECG wire, ECG connector and so on are needed [4]. These are all external connectors, which may lead to pollution of the operating area on the one hand. On the other hand, the external media may affect the stability of the connection and cause errors in the ECG waveform. To improve the accuracy and success rate of puncture, the author designed a new medical guide wire assembly integrating catheter, guide wire and electrode for guiding puncture, which has achieved good efficacy, as reported below.

## Methods

Patients who underwent PICC catheter placement in our hospital from January 2013 to December 2019 were selected as the subjects. Table 1 shows the inclusion and exclusion criteria. These patients were randomly divided into two groups, the observation group and the control group. Random number method is used to group patients. The patient's age, disease, gender, weight, and fall history were recorded. The current study was approved by the Hospital Ethics Committee of the Second Affiliated Hospital of Fujian Medical University. The patient signed the informed consent prior to the puncture. Nurses who perform puncture for patients are specialist nurses with more than 5 years of puncture experience. The nurses were trained and experienced in ultrasound-guided puncture and electrocardiography.

**Table 1:**
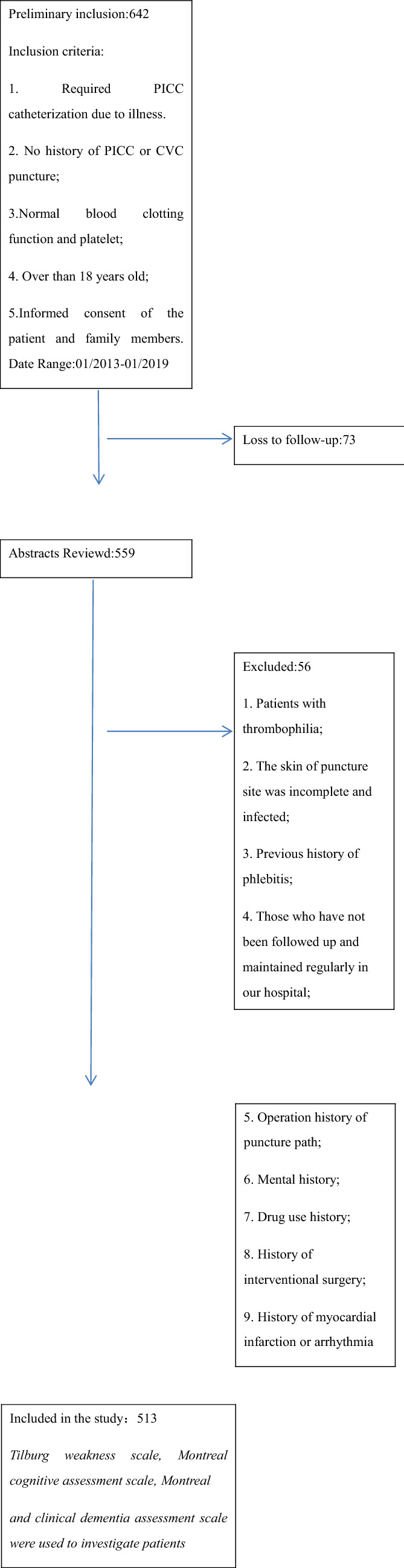
Exclusion criteria and inclusion criteria

The patient was in the supine position. After routine skin cleansing, the nurse connected the patient with ECG monitoring. The basic ECG waveforms of the patient were obtained. The nurse selected the vein to be punctured according to the patient's condition. Generally, basilic vein is selected first. Two-dimensional ultrasound was used to observe the internal diameter, movement, and surrounding anatomical structure of the vein. At the same time, it was also observed whether there were branches and anatomic variations in subclavian vein injection, subclavian vein injection into the indeterminate vein, and whether there were venous valves at the puncture site. Color flow patterns were used to observe and assess the presence of thrombus and flow direction of the vessels. The puncture site was also marked.

**Fig. 1:**
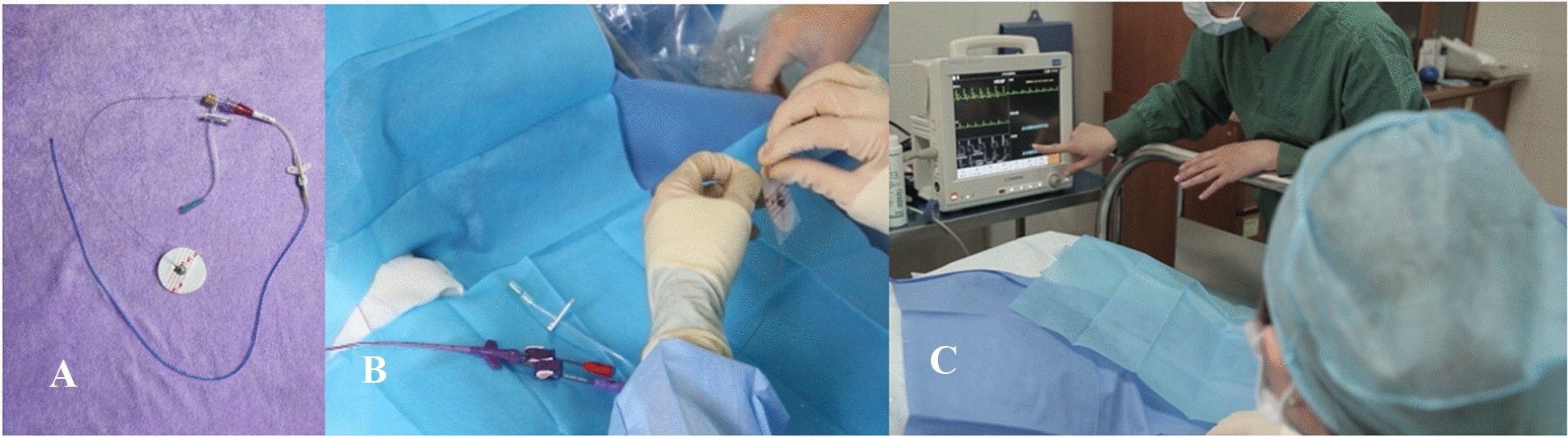
New guide wire is used for puncture. **a** Outline drawing of new guide wire. **b** The integrated design can avoid the contamination of ECG monitoring. **C** ECG monitoring indicated that the waveform was stable and the localization was successful

The patients in the observation group underwent puncture and catheterization guided by the new guide wire. The patient was placed in supine position after taking off the catheter lateral arm clothing. The catheter lateral arm was extended at a 90° angle with the trunk. The puncture exposed the lateral arm and the ipsilateral neck and chest skin. The patient's upper limb length and arm circumference of 10 cm above the elbow were measured as the base value of arm circumference. The length from the pre-puncture point along the vein to the right sternoclavicular joint and back down to the third intercostal space was measured as the length of the preposition tube. The assistant recorded the measurements. The chest area was cleaned and disinfected with 75% ethanol. Three electrodes were attached to the patient's bilateral subclavian and left thoracic major surface skin. ECG monitor was adjusted to II lead. At this point, stable ECG waveforms are used as baseline values for comparison. The arm about to receive the puncture is completely disinfected with iodophor. The puncture user performs vein puncture under the guidance of ultrasound. After seeing blood return, the puncture user delivers the guide wire and exits the puncture needle. Subsequently, the puncturer placed the intubation sheath along the guide wire, withdrew the sheath and guide wire, slowly delivered the catheter to the length of the preset tube along the intubation sheath, and withdrew the intubation sheath. The puncture user connects the premade heparin cap to the PICC supporting guide wire and then inserts the 20 ml physiological saline syringe needle stem into the heparin cap. The puncturer then wipes the blood with 75% ethanol and disinfuses the front end (the softer end) of the saidinga guide wire to the needle stalk of 20 ml normal saline syringe, and delivers the back end (the harder end) of the guide wire to the assistant in a sterile way. Assistant took right clavicle middle of lead electrode (standard II lead right upper limb electrode RA) of patients, in a new electrodes, knot will end after dingle guide who guide wire winding on the electrode slice and tighten, buckle on the electrode RA button loops or clip on electrode RA alligator clip, and ensure good contact. The puncture patient continuously injected 0.9% normal saline into the PICC catheter to open the valve, thus leading to intracardiac ECG. In the process of pipe according to the change of II lead ECG monitor to observe and record the P wave amplitude and guide the position of the catheter placement in the operation, and form the preliminary corrective catheter placement position. Characteristic broad negative P wave and Q wave presented a "W" waveform during intravenous electrocardiogram-guided PICC catheterization, and then the catheter was removed to make the P wave amplitude 50–80% QRS wave amplitude, indicating that the catheter tip reached the lower segment of the superior vena cava, making it an ideal location for PICC catheter tip [5]. When the P wave amplitude is 50–80% QRS wave amplitude, the catheter tip is considered to reach the ideal position. After the tube is placed in place, the Seldinger guide wire is separated and the PICC guide wire synchronously was retracted, the catheter cut according to the actual length of the tube, the connector installed, the positive pressure joint at the end of the catheter connected, the pipe flushed and sealed, and fixed conventionally. After catheterization, the patient underwent chest radiographs to confirm the PICC tip position (See in Fig. 1).

The patients in the control group were punctured with modified MST method after tourniquet ligation. After entering the blood vessel, the catheter was placed under the guidance of traditional guide wire and ECG positioning device.

In the first 2 weeks after puncture, the patients were assessed by specialist nurses every day. The main observations were whether there was blood leakage at the puncture point, whether the tube was blocked, whether the film was curled, whether the infusion connector was loose, whether there was local swelling and heat pain, whether the catheter length was changed, whether the catheter was damaged, etc. When there is swelling of the affected limb, patients need to receive emergency color Doppler ultrasound to exclude thrombosis. The evaluation of phlebitis is based on the classification standard of mechanical phlebitis of intravenous transfusion of Nurses Association [6]. Patients with no clinical manifestations with Grade 0, no phlebitis occurred. When the patient's infusion point showed redness and swelling, but no red line-like changes, and palpation without cord-like changes, the phlebitis was Grade 1. Grade 2 showed a red line-like change on the basis of Grade 1, but there was no linear change on palpation. For Grade 3, changes were found on palpation. On the basis of Grade 3, cordate veins larger than 2.5 cm were palpated in Grade 4 and sometimes pus was discharged.

### Statistical analysis

SPSS statistical package program (SPSS 19.0 version; SPSS Inc., Chicago, IL, USA) was used for statistical analysis. Data were presented as mean ± SD (range), median (range), or *n* (%). χ^2^ test (categorical data) or Student’s *t* test (continuous data) was used to compare the results from two groups. A *P* value of < 0.05 was considered significantly different.

## Results

There was no significant difference in general information between the two groups (see in Table 2). After analyzing the puncture situation of the two groups, it was found that the average catheterization time of the observation group was shorter than that of the control group, and the difference was statistically significant. Patients in the observation group had higher success rate of one-time catheterization and catheterization success rate, and the difference was statistically significant. The incidences of occult thrombosis, phlebitis and catheter blockage in the observation group were lower than those in the control group, and the difference was statistically significant. The incidence of dominant thrombosis and bleeding at the puncture point in the observation group was also lower than that in the control group, but the difference was not statistically significant (see in Table 3).

**Table 2:** General information of two groups of patients

Variable	Observation group (*n* = 206)	Control group (*n* = 311)	Statistical value	*P* value
Gender (male/female)	97/109	152/159	0.16	0.69
The age (years)	59.16 ± 11.36	57.63 ± 9.76	0.10	1.63
BMI	26.35 ± 3.11	25.97 ± 2.95	0.16	1.40
The puncture vein			0.09	0.77
Basilic vein	123	179		
Brachial vein	63	95		
Cephalic vein	20	37		
Catheterization arm (preferred hand/non-preferred hand)	29/177	35/276	0.91	0.34

**Table 3:** Comparison of clinical data between the two groups

Variable	Observation group (*n* = 206)	Control group (*n* = 311)	Statistical value	*P* value
Catheterization time (min)	15.63 ± 7.29	19.15 ± 10.17	4.29	< 0.01
Successful cases of one-time catheterization	203	273	19.66	< 0.01
Successful cases of catheterization	206	302	6.07	0.01
Phlebitis	21	57	6.51	0.01
Dominant thrombus	5	16	2.35	0.13
Occult thrombus	11	29	4.42	0.04
Bleeding at puncture site	15	31	9.25	< 0.01
Catheter blockage	11	29	2.76	0.09

## Discussion

At the same time, a new type of medical guide wire assembly was used to place the tube. The module was designed independently by the author and patented in February 2019. The original intention of the device is to place PICC accurately on the premise of sterility, and to locate the catheter tip accurately by ECG positioning. The successful placement of PICC is of great significance for the treatment of patients [7]. If the ectopic catheter is not found on time, it may lead to serious adverse consequences [8]. To prevent ectopia, many methods such as finger pressing, pressure device blocking and improved posture were used for intraoperative prevention [9]. However, the most important way to prevent catheter displacement is to accurately locate the catheter tip. There are three main positioning methods for PICC catheter: X-ray, DSA and ECG [10]. The former two are seldom used due to radiation. ECG positioning has the advantages of no radiation, no injury and low cost, and this technology can accurately locate the catheter tip position in real time, and has been widely used [11]. Guideline [6] states that if the position of the catheter tip has been confirmed by alternative tip technology, the patient does not need to undergo X-ray and DSA. The traditional ECG positioning technology needs to open the valve and connect the heparin cap, needle, syringe, binding guide wire, binding electrode piece, etc., which takes a long time, and there is a lack of tight connection and serious interference. Sometimes, it cannot lead to continuous, clear and stable ECG waveform, which affects the observation of the puncturer, and may even lead to positioning failure. It is also possible to contaminate the sterile area and increase the incidence of infection. Therefore, the author has developed this kind of guide wire kit which integrates catheter, guide wire, electrode piece and measuring tube, which is expected to realize accurate ECG positioning and heterotopic adjustment of central vascular channel devices such as PICC, central vein, infusion port and hemodialysis catheter. The module is one-piece, with electrode piece, and it is firmly adhered and not easy to shift. The assembly is connected with the pipe screw to ensure the tightness of the connection [12]. In our study, patients in the observation group achieved good ECG waveform and good tip position, and also had a high success rate of puncture and good rate of catheter tip position, which confirms the effectiveness of this technology. The self-contained electrode piece is suitable for most types of ECG monitors and defibrillators, which facilitates the positioning in critically ill patients. The puncture operator can inject or provide intravenous drip of normal saline through the measuring tube provided by the assembly to realize the tip positioning of valve type central vascular access device.

In our study, the observation group had a lower incidence of phlebitis and occult thrombosis, as well as a shorter catheterization time. This may be due to the increased success rate caused by accurate positioning.If the location is not accurate, the vessels are repeatedly mechanically stimulated, which will increase the incidence of phlebitis [13]. At the same time, thanks to our new medical guide wire components, patients are placed under clear ECG monitoring throughout the whole process, and the puncture operator does not need to carry out the tedious steps of connecting the external media many times, and the operation time is greatly reduced.

 It is necessary to study whether this technology can reduce the incidence of thrombosis and whether it can improve the comfort of patients.

## Conclusion

The new type of medical guide wire assembly combined with mobile phone app has definite beneficial effect and accurate positioning. Patients can get higher success rate of one-time catheterization and success rate of catheterization, while the incidence of complications of patients is reduced. This technology is worthy of further promotion.

## Data Availability

The datasets used and/or analyzed during the current study are available from the corresponding author on reasonable request.

## Abbreviations

PICC: Peripherally inserted central venous catheter
